# Supplementary material for: Rationally inattentive intertemporal choice
Source: Nat Commun. 2020 Jul 3;11:3365. doi: 10.1038/s41467-020-16852-y (PMC7335105; doi:10.1038/s41467-020-16852-y)
Supplement: Supplementary file 1 — Reporting Summary [file 41467_2020_16852_MOESM1_ESM.pdf]

nature research

Corresponding author(s): Samuel J Gershman and Rahul Bhui

Last updated by author(s): Apr 27, 2020

Reporting Summary

Nature Research wishes to improve the reproducibility of the work that we publish. This form provides structure for consistency and transparency in reporting. For further information on Nature Research policies, see Authors & Referees and the Editorial Policy Checklist.

Statistics

For all statistical analyses, confirm that the following items are present in the figure legend, table legend, main text, or Methods section.

n/a	Confirmed	
☐	☒	The exact sample size (*n*) for each experimental group/condition, given as a discrete number and unit of measurement
☐	☒	A statement on whether measurements were taken from distinct samples or whether the same sample was measured repeatedly
☐	☒	The statistical test(s) used AND whether they are one- or two-sided<br>*Only common tests should be described solely by name; describe more complex techniques in the Methods section.*
☐	☒	A description of all covariates tested
☐	☒	A description of any assumptions or corrections, such as tests of normality and adjustment for multiple comparisons
☐	☒	A full description of the statistical parameters including central tendency (e.g. means) or other basic estimates (e.g. regression coefficient) AND variation (e.g. standard deviation) or associated estimates of uncertainty (e.g. confidence intervals)
☐	☒	For null hypothesis testing, the test statistic (e.g. *F*, *t*, *r*) with confidence intervals, effect sizes, degrees of freedom and *P* value noted<br>*Give P values as exact values whenever suitable.*
☐	☒	For Bayesian analysis, information on the choice of priors and Markov chain Monte Carlo settings
☒	☐	For hierarchical and complex designs, identification of the appropriate level for tests and full reporting of outcomes
☐	☒	Estimates of effect sizes (e.g. Cohen's *d*, Pearson's *r*), indicating how they were calculated

Our web collection on statistics for biologists contains articles on many of the points above.

Software and code

Policy information about availability of computer code

Data collection	Data was collected on Amazon Mechanical Turk via Turkprime.com, with the task coded in Javascript using jsPsych v6.
Data analysis	Data analysis was conducted using MATLAB R2017B. The code reproducing the analysis is available online at https://github.com/sjgershm/rational-discounting.

For manuscripts utilizing custom algorithms or software that are central to the research but not yet described in published literature, software must be made available to editors/reviewers. We strongly encourage code deposition in a community repository (e.g. GitHub). See the Nature Research guidelines for submitting code & software for further information.

Data

Policy information about availability of data

All manuscripts must include a data availability statement. This statement should provide the following information, where applicable:

- Accession codes, unique identifiers, or web links for publicly available datasets
- A list of figures that have associated raw data
- A description of any restrictions on data availability

The data used in this study are available at https://github.com/sjgershm/rational-discounting.

Field-specific reporting

Please select the one below that is the best fit for your research. If you are not sure, read the appropriate sections before making your selection.

☐ Life sciences ☒ Behavioural & social sciences ☐ Ecological, evolutionary & environmental sciences

Behavioural & social sciences study design

All studies must disclose on these points even when the disclosure is negative.

Study description	This study used a quantitative experimental design.
Research sample	Our experiment comprised 221 adult participants located in the US from Amazon Mechanical Turk (146 males, 67 females, 8 unspecified). Birth decade ranged from the 1940s to the 1990s. This was a convenience sample, and is not necessarily representative of the US or global population. We also re-analyzed the existing data sets of Ballard et al (2017), consisting of 1500 adult participants recruited online from Amazon Mechanical Turk, and Chavez et al (2017), consisting of 1284 Mexican high-school and university students.
Sampling strategy	The sample size for the experiment was determined as comparable to or exceeding typical previous research, such as Hardisty et al. (2013) and Foerde et al. (2016). Similar to work like Hardisty et al., we used a convenience sampling procedure.
Data collection	Participants completed the experiment online on their web browsers. The experiment was programmed in Javascript using jsPsych v6. The researchers were not blind to condition or hypothesis during data collection.
Timing	The data was collected during April 17-23 2019.
Data exclusions	No participants were excluded from the analysis.
Non-participation	Subjects had the option to participate in the study after reading a vague brief description of the task online (which made no specific allusion to intertemporal choice). Subjects were also free to quit at any point during the task. The dropout rate of the task was 6%. No information about the reasons for participant dropout was available.
Randomization	Participants were randomly assigned to each experimental condition within our task software programmed using Javascript and jsPsych.

Reporting for specific materials, systems and methods

We require information from authors about some types of materials, experimental systems and methods used in many studies. Here, indicate whether each material, system or method listed is relevant to your study. If you are not sure if a list item applies to your research, read the appropriate section before selecting a response.

Materials & experimental systems

n/a	Involved in the study	
☒	☐	Antibodies
☒	☐	Eukaryotic cell lines
☒	☐	Palaeontology
☒	☐	Animals and other organisms
☐	☒	Human research participants
☒	☐	Clinical data

Methods

n/a	Involved in the study	
☒	☐	ChIP-seq
☒	☐	Flow cytometry
☒	☐	MRI-based neuroimaging

Human research participants

Policy information about studies involving human research participants

Population characteristics	See above
Recruitment	Participants in our experiment were recruited from Amazon Mechanical Turk, and were required to be above 18 years of age, located in the United States, and to have an "approval rate" (indicating that the participant pays attention and follows instructions correctly in tasks) of at least 98%. While participants self-selected into the experiment due to a combination of monetary incentive and intrinsic motivation to participate in research studies, this population is more diverse in age, race, and socioeconomic status than typical undergraduate research samples (Buhrmester, Kwang, & Gosling, 2011; Perspectives on Psychological Science), and studies have shown that MTurk workers provide high-quality data that replicates many classic findings in experimental psychology (Piolacci & Chandler, 2014; Current Directions in Psychological Science). In addition, we made use of existing data from Chavez et al (2017; JDM) which comprised a large sample of Mexican students. It is not obvious how the predictions of our theory or the properties of the data would be influenced by self-selection.
Ethics oversight	This experiment was approved by the Harvard Committee on the Use of Human Subjects.

Note that full information on the approval of the study protocol must also be provided in the manuscript.
